# Impaired Succinate Oxidation Prevents Growth and Influences Drug Susceptibility in Mycobacterium tuberculosis

**DOI:** 10.1128/mbio.01672-22

**Published:** 2022-07-20

**Authors:** Cara Adolph, Matthew B. McNeil, Gregory M. Cook

**Affiliations:** a Department of Microbiology and Immunology, School of Biomedical Sciences, University of Otago, Dunedin, New Zealand; b Maurice Wilkins Centre for Molecular Biodiscovery, University of Auckland, Auckland, New Zealand; Washington University School of Medicine in St. Louis

**Keywords:** CRISPR interference, *Mycobacterium tuberculosis*, succinate dehydrogenase

## Abstract

Succinate is a major focal point in mycobacterial metabolism and respiration, serving as both an intermediate of the tricarboxylic acid (TCA) cycle and a direct electron donor for the respiratory chain. Mycobacterium tuberculosis encodes multiple enzymes predicted to be capable of catalyzing the oxidation of succinate to fumarate, including two different succinate dehydrogenases (Sdh1 and Sdh2) and a separate fumarate reductase (Frd) with possible bidirectional behavior. Previous attempts to investigate the essentiality of succinate oxidation in M. tuberculosis have relied on the use of single-gene deletion mutants, raising the possibility that the remaining enzymes could catalyze succinate oxidation in the absence of the other. To address this, we report on the use of mycobacterial CRISPR interference (CRISPRi) to construct single, double, and triple transcriptional knockdowns of *sdhA1*, *sdhA2*, and *frdA* in M. tuberculosis. We show that the simultaneous knockdown of *sdhA1 *and* sdhA2* is required to prevent succinate oxidation and overcome the functional redundancy within these enzymes. Succinate oxidation was demonstrated to be essential for the optimal growth of M. tuberculosis, with the combined knockdown of *sdhA1 *and* sdhA2* significantly impairing the activity of the respiratory chain and preventing growth on a range of carbon sources. Moreover, impaired succinate oxidation was shown to influence the activity of cell wall-targeting antibiotics and bioenergetic inhibitors against M. tuberculosis. Together, these data provide fundamental insights into mycobacterial physiology, energy metabolism, and antimicrobial susceptibility.

## INTRODUCTION

Tuberculosis (TB) is a leading cause of infectious disease morbidity and mortality globally, with 10 million new cases and 1.5 million deaths reported in 2020 (1). Successful treatment of infections with Mycobacterium tuberculosis, the causative agent of the disease, requires 6 months of combination therapy involving 2 months of intensive treatment with ethambutol (EMB), isoniazid (INH), rifampicin (RIF), and pyrazinamide (PZA), followed by a further 2 months of treatment with INH and RIF (2). Infections with multidrug-resistant (MDR) and extensively drug-resistant (XDR) strains of M. tuberculosis require up to 2 years of chemotherapy with a combination of 8 to 10 antibiotics and have cure rates as low as 39% for XDR-TB infections (1). Thus, global TB control urgently depends on the development of new drugs and regimens that can quickly and effectively treat both drug-sensitive and drug-resistant TB infections.

Mycobacterial energy generation has emerged as a promising target space for antitubercular drug development. Enzymes within the central carbon metabolism of M. tuberculosis are increasingly being recognized as essential mediators of pathogenicity, and their inhibition or deletion is often bactericidal *in vivo* (3–9). Likewise, a functional respiratory chain is essential for the viability of both replicating and nonreplicating M. tuberculosis (10–14). Several inhibitors of the mycobacterial respiratory chain have now been identified, including the only three new TB drugs to be approved in the last 50 years: bedaquiline (BDQ), which inhibits the mycobacterial F_1_F_o_ ATP synthase (15–17), and pretomanid (PA-824) and delamanid, which result in NO-induced respiratory poisoning (18–22). The clinical efficacy of these respiratory inhibitors (23–26), especially when used in combination (i.e., the recently FDA-approved bedaquiline, pretomanid and linezolid (BPaL) regimen [24, 27, 28]), demonstrates the significant treatment-shortening potential of targeting mycobacterial energy generation.

Succinate oxidation is a major focal point in mycobacterial energy generation and directly couples central carbon metabolism to the respiratory chain (29). The oxidation of succinate to fumarate is catalyzed by succinate dehydrogenase (SDH) enzymes, which couple succinate oxidation in the tricarboxylic acid (TCA) cycle to the reduction of menaquinone in the electron transport chain (ETC) (succinate + menaquinone ↔ fumarate + menaquinol) (30, 31). Fumarate reductase (FRD) enzymes catalyze the reverse reaction: fumarate reduction coupled to menaquinol oxidation. SDH and FRD enzymes encoded by bacteria are highly similar and are often functionally interchangeable (31, 32). M. tuberculosis encodes three different SDH/FRD enzymes which are all predicted to be capable of catalyzing the oxidation of succinate to fumarate (33). This includes two different succinate dehydrogenase enzymes (Sdh1, Rv0249c to Rv0247c, and Sdh2, Rv3316 to Rv3319), as well as a separate fumarate reductase with possible bidirectional behavior (Frd, Rv1552 to Rv1555) (33). All three enzymes have distinct phylogenies, prosthetic groups, and predicted biochemistries (31, 34–37) and are differentially expressed in M. tuberculosis (38–40), suggesting that these enzymes perform distinct, but overlapping, roles.

Previous attempts to elucidate the function of these individual enzymes in M. tuberculosis have focused on the characterization of single-gene deletion mutants (38, 41). However, this leaves the possibility that the remaining enzymes can carry out succinate oxidation in the absence of the other. Consistent with this, single deletion mutants in M. tuberculosis only have minor (i.e., for Δs*dh1* and Δ*sdh2*) (41) or no (i.e., for Δ*frd*) (38) phenotypes, while the construction of double deletion mutants was unsuccessful (41). Similarly, two recent whole-genome CRISPR interference (CRISPRi) studies showed that the depletion of either *sdhA1*, *sdhA2*, or *frdA* alone had no effect on the growth or viability of M. tuberculosis (42, 43). Moreover, while high-throughput transposon hybridization (TraSH) screens have hinted at the essentiality of SDH enzymes for mycobacterial growth and persistence (3, 44, 45), they have also been unable to overcome the functional redundancy in SDH catalysis. Consequently, the essentiality of succinate oxidation in M. tuberculosis is incompletely understood.

Here, we used mycobacterial CRISPR interference (CRISPRi) (46–48) to transcriptionally repress the expression of *sdhA1*, *sdhA2*, and *frdA* in M. tuberculosis alone and in combination, allowing us to overcome the functional redundancy within these enzymes. We show that succinate oxidation is essential for the optimal growth of M. tuberculosis, with the simultaneous knockdown of *sdhA1 *and* sdhA2* (*sdhA1 *+* sdhA2*) significantly impairing the activity of the respiratory chain and preventing growth on a range of carbon sources. Moreover, we demonstrate that impaired succinate oxidation both positively and negatively affects the susceptibility of M. tuberculosis to a variety of anti-TB drugs.

## RESULTS

### Succinate oxidation is essential for the optimal growth of M. tuberculosis.

**FIG 1:**
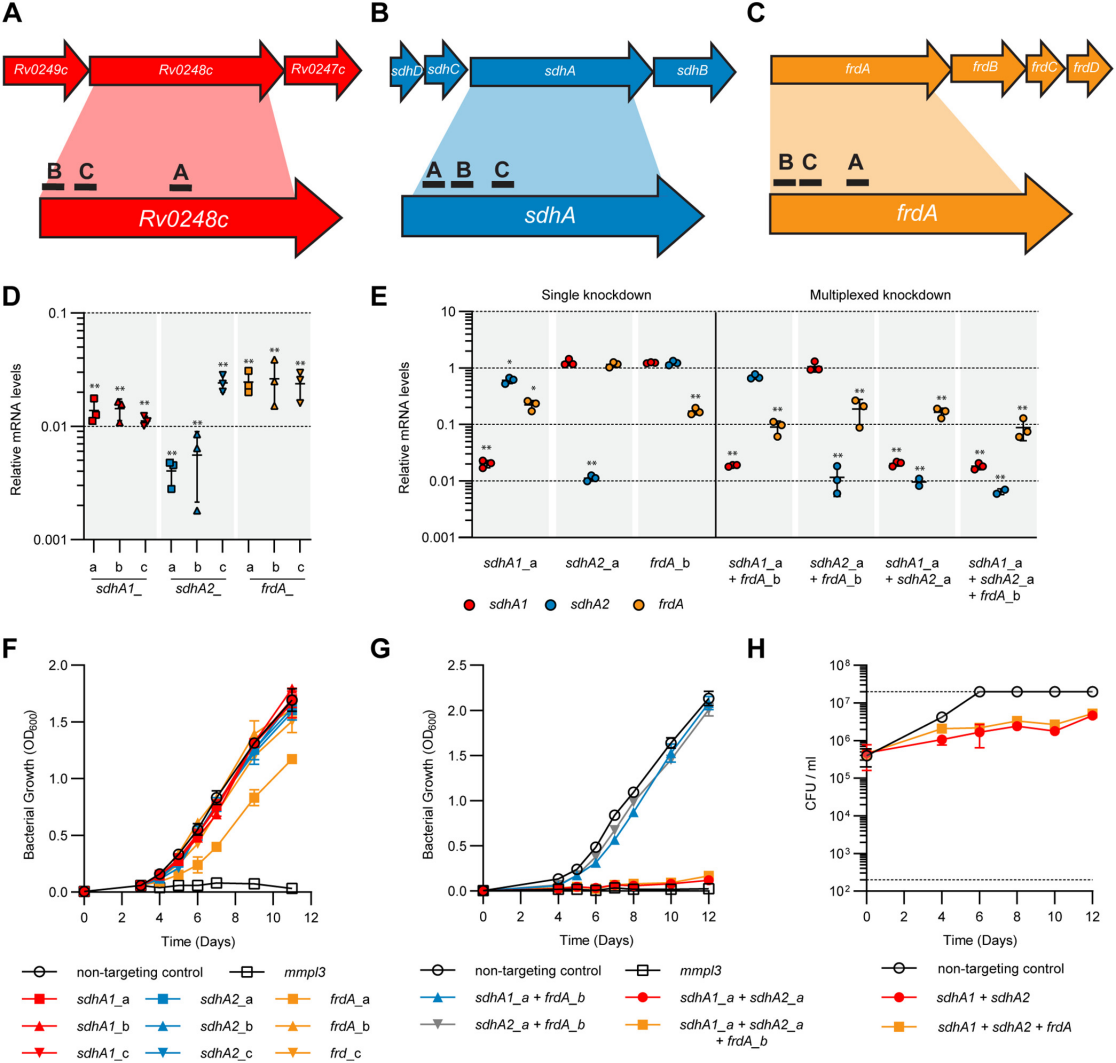
Single and multiplexed transcriptional repression of *sdhA1*, *sdhA2*, and *frdA* in M. tuberculosis using CRISPRi. (A to C) Location of sgRNAs targeting the catalytic subunits of Sdh1 (A), Sdh2 (B), and Frd (C) in M. tuberculosis. sgRNAs were coexpressed with a dCas9_sth1_ under the control of an ATc-inducible promoter. (D) CRISPRi achieves high-level knockdown of target genes in single repression constructs. RNA was harvested 72 h after inducing knockdown (100 ng/mL ATc) and quantified by qPCR. mRNA is expressed relative to a strain expressing a nontargeting control. Results are mean ± standard deviation for three technical triplicates. ** indicates a *P* value of <0.005 from a one-way analysis of variance (ANOVA) with a Dunnett correction comparing each sgRNA to the nontargeting control. (E) CRISPRi achieves high-level knockdown of *sdhA1*, *sdhA2*, and *frdA* in single, double, and triple gene repression constructs. Gene knockdown was quantified and visualized as in panel D. Statistical significance was calculated using a one-way analysis of variance and a Dunnett test for multiple comparisons of the gene expression in each strain against the nontargeting control; *, *P* < 0.01; **, *P* < 0.001. (F) Consequences of the single knockdown of *sdhA1*, *sdhA2*, and *frdA* for the growth of M. tuberculosis in 7H9 medium supplemented with OADC. Knockdown was induced at time zero with ATc (100 ng/mL). An *mmpL3*-targeting sgRNA, which has bactericidal consequences for the viability of M. tuberculosis (48), was included as a positive inhibition control. The means and standard deviation for three replicates are shown. (G and H) Consequences of multiplexed *sdhA1*, *sdhA2*, and *frdA* gene repression for the growth (G) and viability (H) of M. tuberculosis in 7H9 medium supplemented with OADC. Dotted horizontal lines in panel H represent the upper and lower limits of detection of CFU. The means and standard deviation for three replicates are shown. Data in panels G and H are representative of three independent experiments.

To elucidate the roles and essentialities of the SDH and FRD enzymes in M. tuberculosis, we used CRISPR interference (46) to knock down the expression of Sdh1, Sdh2, and Frd alone and in combination. Three guide RNAs (i.e., single guide RNA [sgRNA]) were designed to target the catalytic A subunit of Sdh1, Sdh2, and Frd (Fig. 1A to C), which resulted in high-level transcriptional repression (50- to 100-fold) of target genes in single and multiplexed constructs (Fig. 1D and E). Interestingly, expression of the sgRNA targeting *sdhA1* also resulted in reduced expression of *frdA* in multiplexed repression constructs, regardless of the sgRNA used (Fig. 1E and see also Fig. S1A in the supplemental material), suggesting a shared regulatory mechanism governing *sdh1* and *frd* gene expression.

10.1128/mbio.01672-22.1 FIG S1 Validation of CRISPRi phenotypes using alternative sgRNAs targeting *sdhA1*, *sdhA2*, and *frdA* in M. tuberculosis. (A) Gene knockdown of *sdhA1*, *sdhA2*, and *frdA* in multiplexed repression constructs using one of three different sgRNAs. RNA was harvested 72 h after inducing knockdown (100 ng/mL ATc) and quantified by qPCR. mRNA is expressed relative to a strain expressing a nontargeting control. Results are mean ± standard deviation (SD) for three technical triplicates. *** indicates a *P* value of <0.001 from a one-way analysis of variance (ANOVA) with a Dunnett correction comparing each sgRNA to the nontargeting control. (B and C) Consequences of the double and triple gene repression of *sdhA1 *+* sdhA2 *±* frdA* on the growth (B) and viability (C) of M. tuberculosis in 7H9 medium supplemented with 30 mM succinate. Dashed horizontal lines in panel C represent the upper and lower limits of detection of CFU. The means and standard deviation for three replicates are shown. Download FIG S1, TIF file, 2.4 MB. Copyright © 2022 Adolph et al. 2022 Adolph et al. https://creativecommons.org/licenses/by/4.0/ This content is distributed under the terms of the Creative Commons Attribution 4.0 International license.

Transcriptional repression of either *sdhA1*, *sdhA2*, or *frdA* alone did not affect the growth of M. tuberculosis strain mc^2^6230 in 7H9 medium supplemented with OADC (i.e., with glucose and oleic acid as carbon sources) (Fig. 1F), consistent with the previously published single deletion mutants (38, 41) and transcriptional knockdowns (42, 43). Simultaneous knockdown of both *sdhA1 *+* sdhA2* with or without *frdA* (*sdhA1* + *sdhA2* ± *frdA*) was required to prevent growth in 7H9-OADC medium (Fig. 1G and Fig. S1B) and had bacteriostatic consequences on cell viability (Fig. 1H and Fig. S1C). The combined knockdown of *frdA* with either *sdhA1* or *sdhA2* displayed no growth defect in the same media (Fig. 1G).

**FIG 2:**
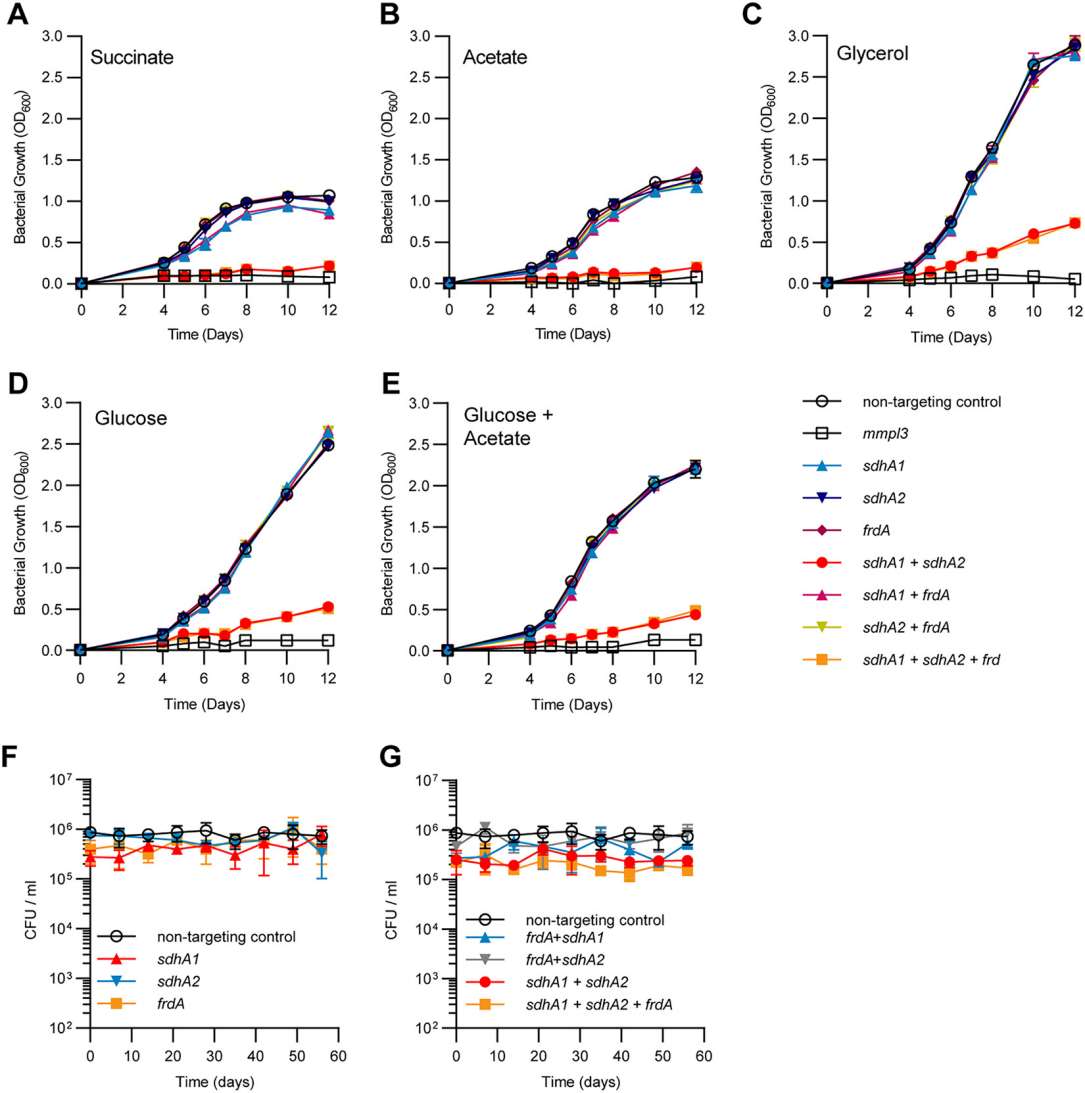
Succinate oxidation is essential for the optimal growth of M. tuberculosis but is dispensable for survival under nutrient starvation. (A to E) Growth profiles of M. tuberculosis single and multiplexed *sdhA1*, *sdhA2*, and *frdA* knockdown strains in 7H9 medium supplemented with 30 mM succinate (A), 0.2% acetate (B), 0.2% glycerol (C), 20 mM glucose (D), or a combination of 0.1% acetate and 10 mM glucose (E). Cultures were grown in 10-mL volumes from a starting OD_600_ of 0.005, and gene knockdown was induced at time zero with ATc (100 ng/mL). An *mmpl3* knockdown strain was included as a positive inhibition control. The means and standard deviation for three replicates are shown. Each experiment was repeated twice. (F and G) Consequences of the single (F) or multiplexed (G) depletion of Sdh1, Sdh2, or Frd for the survival of M. tuberculosis under a nutrient starvation model of nonreplicating persistence. Before entering nutrient starvation, cells were predepleted of SDH and FRD enzymes by inducing transcriptional repression of target genes for 8 days. Cultures were then harvested, washed twice in PBS, and nutrient starved by inoculation into inkwells containing PBS plus tyloxapol. Viability was measured by enumerating CFU per milliliter over 8 weeks. Error bars represent the standard deviation from three replicates.

To further understand the contribution of each enzyme to mycobacterial growth, we investigated the ability of the M. tuberculosis SDH/FRD knockdown strains to grow on a range of fermentable and nonfermentable carbon sources. When provided with succinate as the sole carbon and energy source, the knockdown of *sdhA1* alone, but not *sdhA2* or *frdA*, had a minor (approximately 10%) defect in growth rate (Fig. 2A), consistent with its proposed role as the primary aerobic SDH (41). For all single transcriptional knockdowns, no significant difference in growth compared to a nontargeting control was observed in media containing glucose, glycerol, acetate, or a mixture of glucose and acetate (Fig. 2B to E). This demonstrates that these enzymes are individually dispensable for the growth of M. tuberculosis, regardless of carbon source.

The combined transcriptional repression of *sdhA1 *+* sdhA2* ± *frdA* prevented the optimal growth of M. tuberculosis across all carbon sources tested (Fig. 2A to E). M. tuberculosis repressing *sdhA1 *+* sdhA2* ± *frdA* was unable to grow with the nonfermentable carbon source succinate or acetate as the sole carbon and energy source (Fig. 2A and B). The growth impairment of the *sdhA1 *+* sdhA2* ± *frdA* double or triple knockdown strain was partially rescued when using fermentable carbon sources such as glycerol or glucose, or a combination of acetate and glucose, although growth was still significantly impaired (Fig. 2C to E). Overall, these data demonstrate that a functional SDH enzyme is essential for the optimal growth of M. tuberculosis, regardless of carbon source.

### SDH and FRD enzymes are dispensable for the survival of M. tuberculosis under nutrient starvation.

We next sought to investigate the contribution of SDH and FRD enzymes to the survival of M. tuberculosis under nutrient-starved nonreplicating persistence (39). We first depleted cells of SDH/FRD enzymes by growing strains for 8 days in the presence of anhydrotetracycline (ATc) (100 ng/mL), before placing knockdown strains under nutrient-starved conditions (i.e., phosphate-buffered saline [PBS]). Individual depletion of either Sdh1, Sdh2, or Frd did not affect the survival of M. tuberculosis over 8 weeks of nutrient starvation (Fig. 2F). Moreover, all double and triple transcriptional knockdown strains had no survival defect under nutrient-starved conditions (Fig. 2G). Together, this suggests that SDH and FRD enzymes are dispensable for the survival of M. tuberculosis under nutrient starvation.

### Impaired succinate oxidation significantly disrupts the activity of the mycobacterial respiratory chain.

The oxidation of succinate to fumarate results in two electrons being donated to the ETC (31). Therefore, we sought to determine the bioenergetic consequences of impaired succinate oxidation for the activity of the M. tuberculosis respiratory chain. To achieve this, we predepleted cells of SDH/FRD enzymes by inducing transcriptional repression for 8 days prior to measuring oxygen consumption rates (OCR) as a proxy for respiratory activity. We measured the OCR when cultures were grown with OADC, or with glycerol or succinate as the sole carbon and energy source, as these were the growth conditions under which the SDH/FRD knockdown strains were the least and most impaired, respectively (Fig. 2A and C).

**FIG 3:**
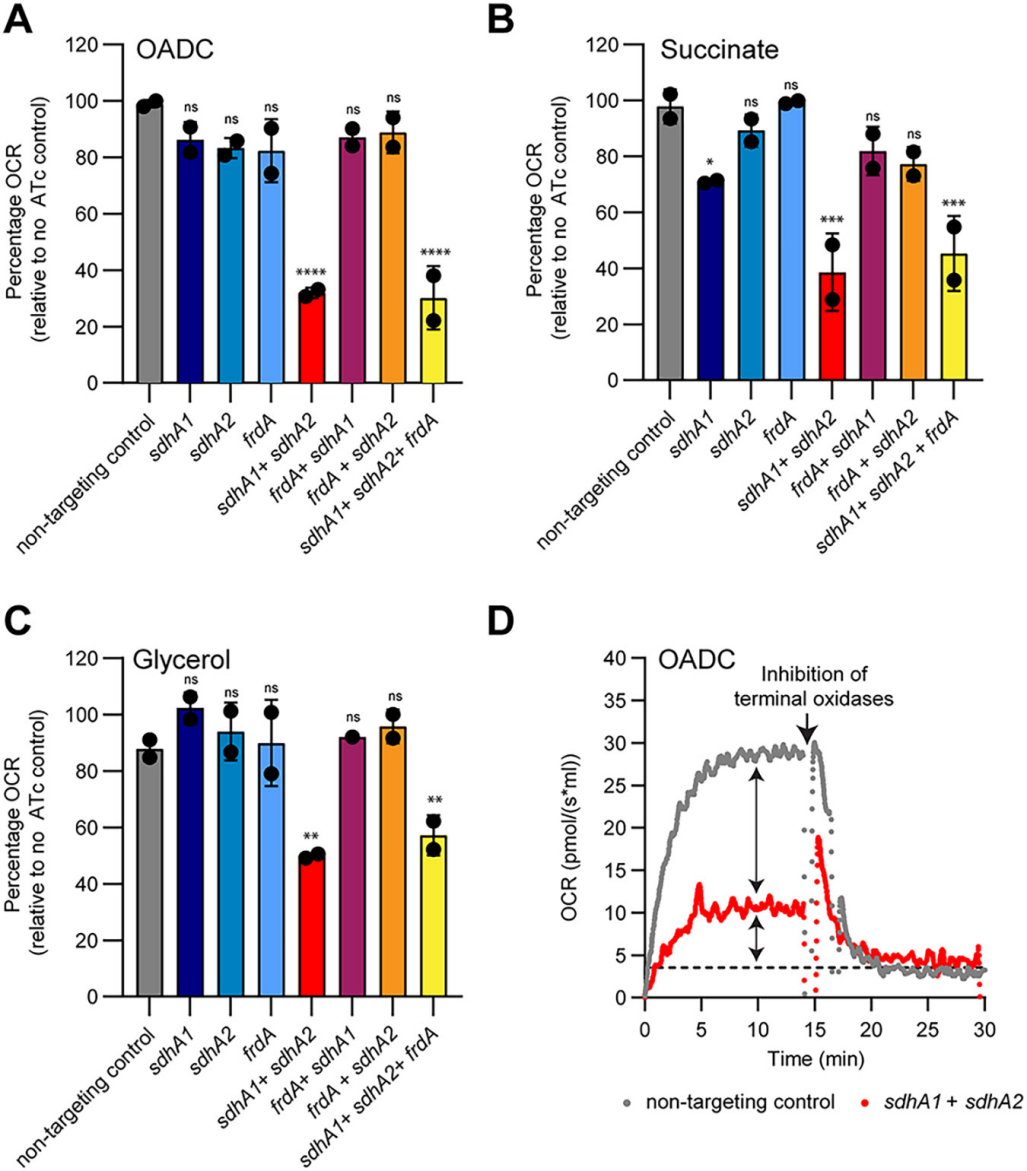
Succinate oxidation is the major contributor of electrons to the respiratory chain in M. tuberculosis. (A to C) Oxygen consumption rates (OCR) of the single and multiplexed *sdhA1*, *sdhA2*, and *frdA* knockdown strains when energized with OADC (A), succinate (B), or glycerol (C). Cultures were grown for 8 days in the presence of 100 ng/mL ATc to deplete cells of SDH/FRD enzymes in 7H9 medium containing the specified carbon sources before performing OCR measurements on cell suspensions. Data were normalized to the respective no-ATc control for each strain. 100% for OADC = ~30.0 pmol/(s × mL), for succinate = ~30.0 pmol/(s × mL), and for glycerol = ~35.0 pmol/(s × mL). Error bars represent the mean and standard deviation for two technical replicates, and data are representative of two independent experiments. Statistical significance was calculated using a one-way analysis of variance and a Dunnett test for multiple comparisons of each strain against the nontargeting control; ns, *P* > 0.05; *, *P* < 0.05; **, *P* < 0.01; ***, *P* < 0.001; and ****, *P* < 0.0001. (D) Chemical inhibition of both terminal oxidases with Q203 (400 nM) and ND-011992 (100 μM) demonstrates that succinate oxidation is the major contributor of electrons to the respiratory chain. Cultures were grown for 8 days in the presence of 100 ng/mL ATc to deplete cells of SDH/FRD enzymes in 7H9-OADC medium before performing OCR measurements on cell suspensions. Dashed horizonal lines in panel D represent the baseline OCR. Arrows represent the contribution of succinate oxidation to the OCR of M. tuberculosis and the residual OCR in the absence of succinate oxidation, respectively. Data are representative of two independent experiments.

When grown in media supplemented with OADC, only the combined knockdown of *sdhA1 *+* sdhA2* was able to overcome the functional redundancy in succinate oxidation and affect the OCR of M. tuberculosis (Fig. 3A). All other combinations of transcriptional repression did not influence the OCR of M. tuberculosis when grown with OADC (Fig. 3A). In contrast, with succinate as the sole carbon source, the knockdown of *sdhA1* alone reduced the OCR of M. tuberculosis by approximately 20% (Fig. 3B). This is consistent with the growth impairment of this strain when grown on succinate (Fig. 2A) and suggests that Sdh1 is the dominant SDH enzyme in M. tuberculosis. No significant difference in OCR was observed for the *sdhA1* knockdown strain in media supplemented with glycerol (Fig. 3C).

The combined repression of *sdhA1 *+* sdhA2 *±* frdA* significantly reduced the respiration rate of M. tuberculosis across all three carbon sources tested (Fig. 3A to C). The double and triple *sdhA1* + *sdhA2* ± *frdA* knockdown strains had a 50% reduction in OCR compared to their respective no-ATc controls in media containing glycerol and a 70% reduction in OCR when energized with OADC or succinate (Fig. 3A to C). To further investigate the contribution of succinate oxidation to total respiratory flux, we used Q203 and ND-011992 to inhibit the activity of both terminal oxidases (cytochrome *bc*_1_:*aa*_3_ and cytochrome *bd*, respectively) and abolish respiration (49, 50). The complete inhibition of aerobic respiration highlighted an additional ~30% respiratory capacity in the endogenous respiration rate of M. tuberculosis in the absence of succinate oxidation (Fig. 3D). Combined, these results demonstrate that succinate oxidation is the master driver of M. tuberculosis respiration, regardless of carbon source. This may occur directly (i.e., through succinate being the major contributor of electrons to the ETC) or indirectly through SDH activity regulating the activity of the TCA cycle.

### Impaired succinate oxidation influences the drug susceptibility of M. tuberculosis.

TB is treated with multidrug therapies, and as such, any new drug needs to be effective in combination. While bioenergetic inhibitors are proving promising at reducing treatment times, the consequences of inhibiting respiration for the activity of other antibiotics are not clearly understood. Several studies have linked antibiotic killing to the dysregulation of respiration and the production of reactive oxygen species (ROS) (51–57), while others have demonstrated that inhibiting respiration can attenuate the activity of bactericidal drugs (56, 58–62). Given that impaired succinate oxidation reduced the activity of the respiratory chain by approximately 70% (Fig. 3), we sought to investigate how the simultaneous knockdown of *sdhA1 *+* sdhA2* affected the activity of anti-TB drugs against M. tuberculosis. We profiled the *sdhA1 *+* sdhA2* double knockdown strain of M. tuberculosis against TB drugs with a range of cellular targets including cell wall biosynthesis (INH, PA-824, ethionamide [ETH], EMB, and SQ109), DNA replication (levofloxacin [LEV]), transcription (RIF), protein synthesis (linezolid [LZD] and streptomycin [STREP]), and bioenergetics (BDQ, clofazimine [CFZ], thioridazine [THZ], SQ109, Q203, TB47, and PA-824). We induced gene knockdown and treated cultures with antibiotics simultaneously on day 0 as previously described (47, 48) and monitored killing (rather than MIC) as a measure of susceptibility as M. tuberculosis is significantly impaired for growth when transcriptionally repressing *sdhA1 *+* sdhA2* (Fig. 1 and 2).

**FIG 4:**
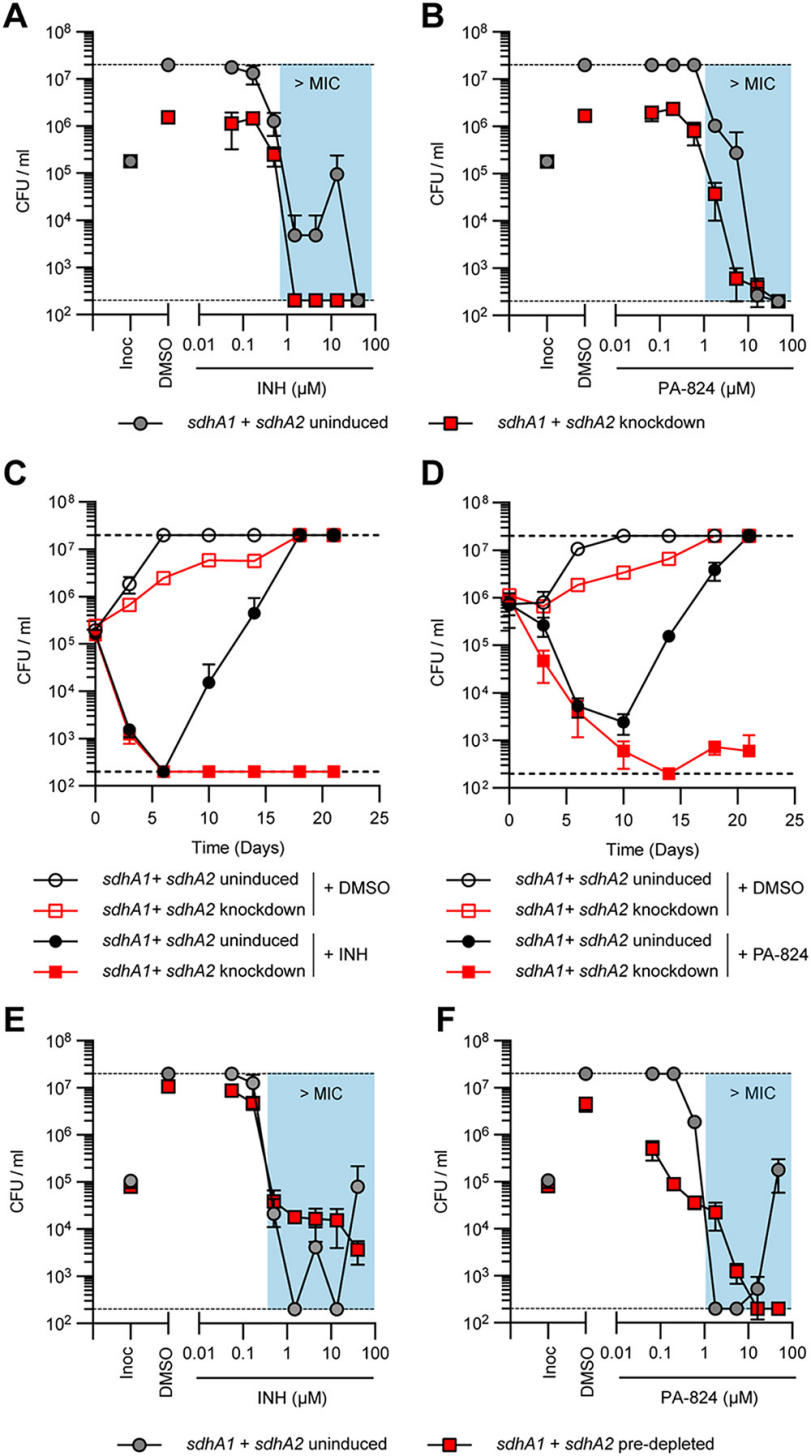
Impaired succinate oxidation by the joint transcriptional repression of *sdhA1 *and* sdhA2* alters INH and PA-824 susceptibility and resistance in M. tuberculosis. (A and B) Effect of the transcriptional repression of *sdhA1 *and* sdhA2* on the susceptibility of M. tuberculosis to INH (A) and PA-824 (B) when gene knockdown was induced simultaneously with antibiotic challenge. Cultures were grown in 96-well plates from a starting OD_600_ of 0.005 with 0 or 100 ng/mL ATc and a 7-point, 3-fold dilution gradient of each drug. Viability was determined after 10 days of incubation, and CFU per milliliter was enumerated after 5 weeks. Results are means and standard deviations from three replicates and are representative of three independent experiments. (C and D) Viability of M. tuberculosis *sdhA1 *+* sdhA2* knockdown strains treated with INH (C) or PA-824 (D). Cultures were grown in 7H9-OADC-PAN-KAN medium in 10-mL volumes from a starting OD_600_ of 0.005. Knockdown was induced with 100 ng/mL ATc on day 0, and CFU per milliliter was determined on stated days. INH was used at 13.5 μM and PA-824 at 1.8 μM. Results are the mean and standard deviation for three replicates and are representative of three experiments. (E and F) Susceptibility of M. tuberculosis transcriptionally repressing *sdhA1 *and* sdhA2* to INH (E) and PA-824 (F) when cells were predepleted of SDH enzymes prior to antibiotic challenge. Cultures were treated with 0 or 100 ng/mL ATc for 6 days to deplete cells of SDH enzymes, before harvesting cells and inoculating them into 96-well plates containing a 7-point, 3-fold dilution gradient of each drug and 0 or 100 ng/mL ATc at a starting OD_600_ of 0.005. Viability was determined by plating for CFU per milliliter after a further 10 days of incubation. Results are the means and standard deviations for three replicates and are representative of three independent experiments. Blue boxes in panels A, B, E, and F denote concentrations above the MIC of the no-knockdown control. Dashed horizontal lines represent the upper and lower levels of detection. Inoc, CFU per milliliter at inoculation (i.e., time = 0).

The combined knockdown of *sdhA1 *+* sdhA2* resulted in increased killing of M. tuberculosis by INH and PA-824 after 10 days of incubation (Fig. 4A and B), while the susceptibility to all other drugs tested remained unchanged (Fig. S2). For example, concentrations above 1.5 μM INH resulted in a 3-log_10_ reduction in CFU per milliliter to below the limit of detection for the *sdhA1 *+* sdhA2* double knockdown strain, whereas in the no-knockdown control the same level of killing was achieved only at the highest concentration tested (40.5 μM INH) (Fig. 4A). Note that the large “jump” in CFU per milliliter for the no-knockdown control at 13.5 μM INH was due to the emergence of INH resistance in one well. Similarly, 1.8 μM and 5.4 μM PA-824 resulted in a 0.6- and 2.5-log_10_ reduction in CFU per milliliter, respectively, when M. tuberculosis was repressing *sdhA1 *+* sdhA2*, but neither concentration killed the no-knockdown control (Fig. 4B). Combined, these data suggest that impaired succinate oxidation synergizes with INH and PA-824 when knockdown is induced simultaneously with antibiotic challenge.

10.1128/mbio.01672-22.2 FIG S2 Impact of impaired succinate oxidation on the activity of various TB drugs against M. tuberculosis. Effect of the simultaneous transcriptional repression of *sdhA1 *and* sdhA2* on the susceptibility of M. tuberculosis to various TB drugs. Cultures were grown in 96-well plates from a starting OD_600_ of 0.005 with a 7-point, 3-fold dilution gradient of each drug. Gene knockdown was induced on day 0 with 0 or 100 ng/mL ATc. Viability was determined after 10 days of incubation. Blue boxes denote concentrations above the MIC of the no-knockdown control. Dashed horizontal lines represent the upper and lower levels of detection. Results are the mean and standard deviation for two replicates. Abbreviations: EMB, ethambutol; ETH, ethionamide; BDQ, bedaquiline; CFZ, clofazimine; THZ, thioridazine; RIF, rifampicin; STREP, streptomycin; LZD, linezolid; LEV, levofloxacin; Inoc, CFU per milliliter at inoculation (i.e., time = 0). Download FIG S2, TIF file, 2.6 MB. Copyright © 2022 Adolph et al. 2022 Adolph et al. https://creativecommons.org/licenses/by/4.0/ This content is distributed under the terms of the Creative Commons Attribution 4.0 International license.

We investigated this interaction further by monitoring killing by INH and PA-824 over time. Interestingly, there was no change in the rate of killing by either drug, but instead the simultaneous knockdown of *sdhA1 *+* sdhA2* prevented the emergence of resistance to both INH and PA-824 (Fig. 4C and D). After 3 weeks, no INH- or PA-824-resistant mutants were isolated when both Sdh1 and Sdh2 were depleted, while INH and PA-824 resistance emerged by day 10 in the no-knockdown controls (Fig. 4C and D). This could indicate that normal respiratory chain function is required for the development of INH and PA-824 resistance as previously described (14, 63, 64), or it could be the result of the reduced replication rate of M. tuberculosis when repressing *sdhA1 *+* sdhA2.* Either way, these results demonstrate that preventing the emergence of INH and PA-824 resistance underlies the synergy observed at day 10 (Fig. 4A and B).

### Predepletion of SDH enzymes reveals additional interactions with cell wall inhibitors and bioenergetic inhibitors.

Interestingly, and in contrast to our results, impaired respiration through inhibition of the cytochrome *bc*_1_:*aa*_3_ terminal oxidase or ATP synthase has previously been shown to antagonize INH activity (58–60). To address this discrepancy, we considered whether depleting cells of CRISPRi target enzymes prior to antibiotic challenge affected the interactions observed. We exploited the known synthetic lethality between the two terminal oxidases (the cytochrome *bc*_1_:*aa*_3_ complex and cytochrome *bd*) (13, 49, 65, 66) as a positive control. To do this, we predepleted M. tuberculosis of the cytochrome *bd* oxidase using CRISPRi for 6 days before treating cultures with the *bc*_1_ inhibitors Q203 and TB47 (67) which are bactericidal against cells lacking a functional cytochrome *bd* but bacteriostatic against wild-type (WT) cells (13, 49, 65, 66). Killing of M. tuberculosis was observed only when cells were predepleted of cytochrome *bd*, resulting in cell death up until day 22 (the endpoint of the experiment), but not when knockdown was induced simultaneously with antibiotic challenge (Fig. S3). Given the prolonged time frame of this experiment and the propensity for suppressor mutants when using ATc-inducible systems (48), we further confirmed the sustained knockdown of CRISPRi target enzymes when using this experimental setup by demonstrating that the predepleted *sdhA1 *+* sdhA2* strain was inhibited for growth over 10 days to a similar level as that when gene knockdown was induced on day 0 (Fig. S4). Taken together, these results demonstrate that there is a requirement for the predepletion of CRISPRi target enzymes when screening for chemical-genetic interactions in M. tuberculosis and show that target depletion is maintained over long time periods at sufficient levels to detect interactions (68).

10.1128/mbio.01672-22.3 FIG S3 Impact of predepleting cells of CRISPRi target enzymes before antibiotic challenge. (A) Susceptibility of M. tuberculosis transcriptionally repressing cytochrome *bd* to Q203 (400 nM) and TB47 (400 nM) when knockdown was induced with 100 ng/mL ATc at day 0. (B) Susceptibility of M. tuberculosis transcriptionally repressing cytochrome *bd* to Q203 (400 nM) and TB47 (400 nM) when cells were predepleted of cytochrome *bd* by inducing gene knockdown (100 ng/mL ATc) for 6 days prior to antibiotic challenge. Cultures were grown in 7H9-OADC-PAN-KAN medium in 10-mL volumes from a starting OD_600_ of 0.005. CFU per milliliter was determined on stated days and enumerated after 3 weeks of incubation. Dashed horizontal lines represent the upper and lower limits of detection. Results are the means and standard deviations from two replicates. Download FIG S3, TIF file, 2.8 MB. Copyright © 2022 Adolph et al. 2022 Adolph et al. https://creativecommons.org/licenses/by/4.0/ This content is distributed under the terms of the Creative Commons Attribution 4.0 International license.

10.1128/mbio.01672-22.4 FIG S4 Impact of predepleting cells of SDH enzymes on the growth of M. tuberculosis. Gene knockdown was induced with 100 ng/mL ATc either on day 0 (blue) or for 6 days prior (red) before inoculating cells into fresh 7H9 medium supplemented with OADC-PAN-tyloxapol-KAN and 100 ng/mL ATc at a starting OD_600_ of 0.005. Growth was monitored until day 10 (endpoint of antibiotic susceptibility screening assays) by measuring the OD_600_ every 2 days. An *mmpl3*-targeting sgRNA, which has bactericidal consequences for the viability of M. tuberculosis (48), was included as a positive inhibition control. Results are the means and standard deviations from two replicates. Download FIG S4, TIF file, 1.2 MB. Copyright © 2022 Adolph et al. 2022 Adolph et al. https://creativecommons.org/licenses/by/4.0/ This content is distributed under the terms of the Creative Commons Attribution 4.0 International license.

**FIG 5:**
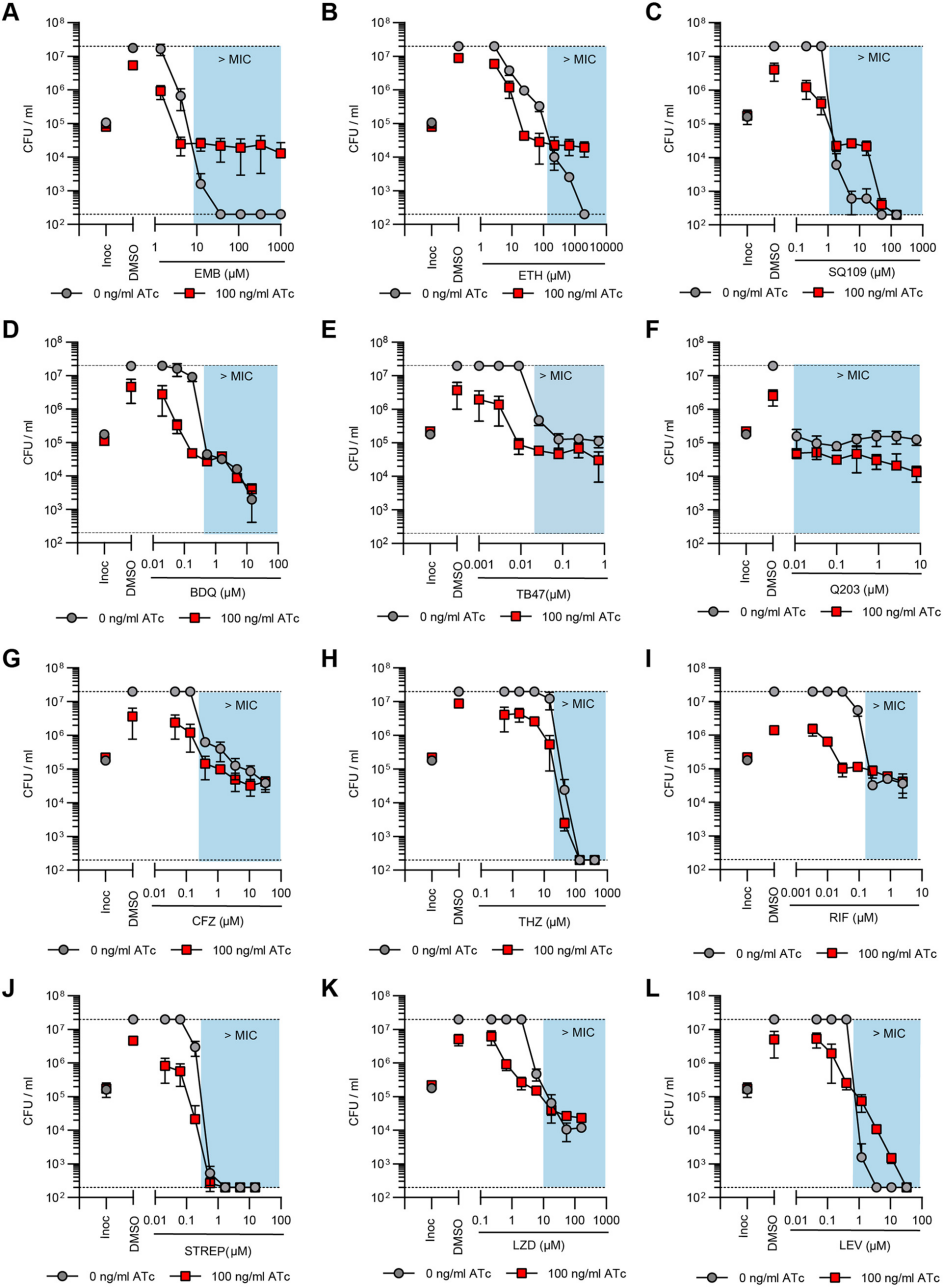
Impaired succinate oxidation by the dual depletion of Sdh1 and Sdh2 synergizes with bioenergetic inhibitors in M. tuberculosis but attenuates the activity of cell wall inhibitors. (A to C) Effect of the joint transcriptional repression of *sdhA1 *and* sdhA2* on the susceptibility of M. tuberculosis to the cell wall inhibitors ethambutol (EMB) (A), ethionamide (ETH) (B), and SQ109 (C). (D to H) Effect of the joint transcriptional repression of *sdhA1 *and* sdhA2* on the susceptibility of M. tuberculosis to bioenergetic inhibitors: BDQ (D), TB47 (E), Q203 (F), clofazimine (CFZ) (G), and thioridazine (THZ) (H). (I to L) Effect of the joint transcriptional repression of *sdhA1 *and* sdhA2* on the susceptibility of M. tuberculosis to inhibitors of transcription, translation, or DNA replication: rifampicin (RIF) (I), streptomycin (STREP) (J), linezolid (LZD) (K), and levofloxacin (LEV) (L). Cultures were predepleted of SDH enzymes by inducing gene knockdown (0 or 100 ng/mL ATc) for 6 days and then inoculated into 96-well plates at a starting OD_600_ of 0.005 with 0 or 100 ng/mL ATc and a 7-point, 3-fold dilution gradient of each drug. Viability was determined after 10 days of incubation, and CFU per milliliter was enumerated after 5 weeks. Blue boxes denote concentrations above the MIC of the no-knockdown control. Dashed horizontal lines represent the upper and lower levels of detection. Results are the mean and standard deviation from three replicates and are representative of at least two independent experiments. Inoc, CFU per milliliter at inoculation (i.e., time = 0).

Consequently, we predepleted M. tuberculosis of SDH enzymes by inducing CRISPRi for 6 days before challenging cultures with the TB drugs listed above. Under these conditions, the dual depletion of Sdh1 and Sdh2 attenuated the bactericidal activity of INH against M. tuberculosis at concentrations above the MIC, while no difference in susceptibility was seen at concentrations below the MIC (Fig. 4E). As in Fig. 4A, the large “jumps” in CFU per milliliter for the no-knockdown control were due to the emergence of INH resistance in one well. In contrast, depletion of Sdh1 and Sdh2 sensitized M. tuberculosis to growth inhibition by concentrations of PA-824 that were below the MIC of the no-knockdown control (i.e., 0.06 to 0.6 μM PA-824) but, conversely, attenuated the bactericidal activity of PA-824 at concentrations above the MIC (i.e., 1.8 and 5.4 μM PA-824) (Fig. 4F). Interestingly, this attenuation was overcome at higher concentrations of PA-824 (i.e., 16.2 and 48.6 μM PA-824), resulting in the sterilization of cultures and preventing the emergence of resistance seen in the no-knockdown control (Fig. 4F). As with INH, the depletion of Sdh1 and Sdh2 attenuated the activity of all three other cell wall inhibitors tested: EMB, ETH, and SQ109 (Fig. 5A to C). Combined, these data demonstrate that impaired succinate oxidation attenuates the bactericidal activity of cell wall inhibitors against M. tuberculosis when cells are first depleted of SDH enzymes.

In contrast, the dual depletion of Sdh1 and Sdh2 increased the susceptibility of M. tuberculosis to the bioenergetic inhibitors BDQ, TB47, and Q203, which target the ATP synthase and cytochrome *bc*_1_:*aa*_3_ terminal oxidase, respectively (Fig. 5D to F). M. tuberculosis with both Sdh1 and Sdh2 depleted was hypersusceptible to growth inhibition and/or killing by BDQ at concentrations that were below the MIC of the no-knockdown control (i.e., 0.06 μM BDQ prevented the growth of the SDH-depleted cultures and 0.18 μM BDQ resulted in an ~0.5-log_10_ reduction in CFU per milliliter while neither concentration affected the growth of the no-knockdown control) (Fig. 5D). Likewise, concentrations of TB47 that were below the MIC of the no-knockdown control were bacteriostatic against M. tuberculosis when Sdh1 and Sdh2 were depleted, while concentrations above the MIC (i.e., 0.1 to 1 μM TB47) resulted in an ~0.5- to 0.9-log_10_ reduction in CFU per milliliter in SDH-depleted cells compared to the bacteriostatic effects on WT cells (Fig. 5E). Moreover, the depletion of Sdh1 and Sdh2 rendered M. tuberculosis susceptible to killing by the normally bacteriostatic Q203, resulting in a 1.2-log_10_ reduction in CFU per milliliter at the highest concentration tested (Fig. 5F). Depletion of Sdh1 and Sdh2 also resulted in slight increases in susceptibility to the two other bioenergetic inhibitors tested, CFZ and THZ, at concentrations around the MIC of the respective compound, although no differences in killing were observed above the MIC (Fig. 5G and H). Taken together, these findings demonstrate that impaired succinate oxidation through the depletion of Sdh1 and Sdh2 broadly synergizes with other bioenergetic inhibitors in M. tuberculosis.

Further, impaired succinate oxidation synergized with RIF, LZD, and STREP at sub-MICs with the depletion of Sdh1 and Sdh2, resulting in the growth inhibition or killing of M. tuberculosis at concentrations that do not affect the growth of the no-knockdown control (Fig. 5I to K). However, there were no differences in the magnitude of killing above the respective MIC of each compound (Fig. 5I to K). Finally, as with the cell wall inhibitors, impaired succinate oxidation attenuated the bactericidal activity of the fluroquinolone antibiotic LEV, resulting in reduced killing against M. tuberculosis with Sdh1 and Sdh2 depleted (Fig. 5L).

## DISCUSSION

Succinate oxidation is a major focal point in both the central carbon metabolism and respiratory chain of M. tuberculosis, and yet its essentiality remains poorly characterized. Here, we address this by reporting on the roles and essentialities of three different enzymes (Sdh1, Sdh2, and Frd) predicted to be capable of catalyzing the oxidation of succinate to fumarate in M. tuberculosis and on the consequences of impaired succinate oxidation. We show that Sdh1 is the primary aerobic SDH utilized by M. tuberculosis but that the simultaneous transcriptional repression of both *sdhA1 *and* sdhA2* is required to overcome the functional redundancy within the SDH/FRD enzymes and prevent growth. Our results demonstrate that succinate oxidation is the major contributor of electrons to the respiratory chain and is required for the optimal growth of M. tuberculosis on both fermentable and nonfermentable carbon sources. Finally, we show that impaired succinate oxidation both positively and negatively affects the susceptibility of M. tuberculosis to a variety of anti-TB drugs.

Sdh1 has previously been proposed to be the primary aerobic SDH in M. tuberculosis, with its deletion resulting in a minor growth impairment in media containing succinate as the sole carbon and energy source (41). Likewise, the transcriptional repression of *sdhA1* impaired the growth and OCR of M. tuberculosis in succinate-containing media, validating our CRISPRi approach to studying the essentiality of SDH and FRD enzymes in M. tuberculosis. Notably, the deletion of *sdh1* was reported to result in an increased rate of respiration in M. tuberculosis (41), which may appear to contradict our finding that the single *sdhA1* knockdown strain consumes oxygen more slowly in succinate-containing media. However, the previously described increased respiration rate in the *Δsdh1* strain was observed only when oxygen levels fell below ~40% dissolved oxygen, while oxygen consumption rates were similar at higher oxygen tensions (41). In our experiments using the same media (containing glycerol as an energy source), we observed no difference in OCR between the single *sdhA1* knockdown strain and the no-knockdown control, thus reconciling the two findings.

In addition to validating Sdh1 as the primary aerobic SDH in M. tuberculosis, our work elucidates some of the compensatory mechanisms within succinate oxidation by demonstrating that Sdh2 can catalyze succinate oxidation in the absence of Sdh1. However, the growth and oxygen consumption impairments observed when Sdh2 is the only functional SDH enzyme present (i.e., when *sdhA1 *and* frdA* are transcriptionally repressed) suggest that Sdh2 is less efficient at catalyzing succinate oxidation than Sdh1. The molecular mechanisms underlying this are unclear, although both enzymes appear to use different reaction mechanisms to drive succinate oxidation (35–37). Moreover, these findings are in contrast to Mycobacterium smegmatis, where Sdh2 is proposed to have a higher affinity for succinate oxidation and is essential, while Sdh1 can be deleted or transcriptionally silenced with no identifiable phenotype (34, 69).

We noted that the transcriptional repression of *sdhA1* resulted in a concomitant reduction in *frdA* expression in single and multiplexed repression constructs, regardless of the sgRNA used, suggesting that a shared regulatory mechanism governs *sdh1* and *frd* gene expression in M. tuberculosis. Despite this, no difference in *frdA* expression was observed in the *Δsdh1* strain (41), meaning that this finding requires additional investigation. Most significantly, the linked repression of *sdhA1* and *frdA* made it difficult to rule out a role for Frd in M. tuberculosis succinate oxidation as Sdh1 and Sdh2 could not be depleted independently of Frd. Therefore, additional experimentation is required to determine whether Frd contributes to succinate oxidation in M. tuberculosis. Previous bioinformatic analysis suggests that the M. tuberculosis Frd enzyme contains the residues required to covalently bind a flavin adenine dinucleotide (FAD) cofactor [including the conserved histidine needed to make the 8α-*N*(3)-histidyl linkage] (70), which is required for succinate oxidation (70–73). However, the high degree of similarity between SDH and FRD enzymes (including at the FAD site) means that this is insufficient evidence to predict the reaction(s) catalyzed. Moreover, even if Frd is capable of oxidizing succinate, bacterial FRD enzymes are a major source of ROS (74–77), meaning that the utilization of Frd aerobically to drive succinate oxidation may be detrimental to M. tuberculosis. Frd is upregulated >200-fold under hypoxia (38), and we therefore suggest that Frd predominately functions in the reverse TCA cycle, which is essential for survival under hypoxia (3, 38, 78), rather than contributing to aerobic succinate oxidation. However, this still requires experimental validation.

While individually dispensable, the dual depletion of both Sdh1 and Sdh2 demonstrates the essentiality of succinate oxidation in M. tuberculosis. The double *sdhA1 *+* sdhA2* knockdown strain was significantly impaired for growth across a range of carbon sources, including the fermentable carbon sources glycerol and glucose, as well as the nonfermentable carbon sources succinate and acetate. The improved growth on glucose and glycerol is likely due to substrate-level phosphorylation augmenting ATP levels. Importantly, mycobacterial growth and persistence *in vivo* are believed to be fueled by fatty acids (4, 40, 79), rather than glycerol and glycolytic carbon sources (80). Thus, the inability of M. tuberculosis to grow on acetate (a fatty acid surrogate [78]) when succinate oxidation is impaired suggests that inhibition of SDH activity may prevent growth *in vivo*. This is supported by the previously reported survival defect of the M. tuberculosis Δ*sdh1* strain in mouse lungs (41). However, this needs to be investigated further using the double *sdhA1 *+* sdhA2* knockdown strain in M. tuberculosis infection models.

The simultaneous knockdown of *sdhA1 *and* sdhA2* reduced the OCR of M. tuberculosis by approximately 70%, demonstrating that succinate oxidation is essential for the normal function of the respiratory chain. This is interesting as the ETC of M. tuberculosis is known for its significant plasticity, with the bacterium encoding multiple other primary dehydrogenases in addition to its two SDH enzymes (33, 81) that could theoretically compensate for reduced SDH activity. The simplest explanation for the observed reduction in OCR is that the majority of electrons are donated to the respiratory chain of M. tuberculosis through the oxidation of succinate to fumarate. However, NADH is generally considered the primary electron donor for aerobic growth in mycobacteria (81). Therefore, it is possible that the reduced OCR in the double *sdhA1 *+* sdhA2* knockdown strain is reflective of an overall reduction in central carbon metabolism and respiration to circumvent the loss of SDH activity. Succinate oxidation appears to be an invariant component of mycobacterial central carbon metabolism with few options to reroute around it (29), and we hypothesize that cells downregulate metabolism and respiration to prevent an accumulation of succinate, which may disrupt the metabolic homeostasis of replicating cells. Regardless, our data support the previous proposal that SDH is the master regulator of respiration in M. tuberculosis (41).

The essentiality of succinate oxidation for the optimal growth and ETC activity of M. tuberculosis complements previous reports on the requirement for SDH/FRD enzymes for mycobacterial persistence (3, 34, 38, 41, 44, 78). While not investigated in this study due to technical limitations of using CRISPRi under hypoxia, several studies have reported the essentiality of succinate metabolism for the survival of M. tuberculosis under low oxygen tensions (3, 34, 38, 41, 44, 78). In particular, inhibition of SDH activity using the suicide inhibitor 3-nitropropionate (3-NP) resulted in time-dependent killing of M. tuberculosis during adaptation to hypoxia (78). In contrast, our results suggest that the mycobacterial SDH/FRD enzymes are dispensable for survival under nutrient starvation, despite Frd being strongly upregulated under this condition (39). However, a potential limitation of using transcriptional repression without concomitant protein degradation (82, 83) is that there may be residual SDH/FRD enzymes in the cell that could catalyze succinate oxidation. This is particularly relevant under nonreplicating conditions (i.e., nutrient starvation) where the lower energetic requirement of cells (i.e., maintenance energy) (10, 11) means that even a small amount of residual enzyme may be sufficient to sustain persistence. Therefore, while our data argue against a role for SDH/FRD under nutrient starvation, this needs additional validation.

In addition to providing fundamental insights into the physiology of mycobacterial energy metabolism, the findings described in this study add to the complex narrative surrounding the interaction between modulating respiration and antimicrobial efficacy. Here, we showed that inhibition of succinate oxidation through the depletion of Sdh1 and Sdh2 attenuated the bactericidal activity of several cell wall inhibitors against M. tuberculosis, including INH, ETH, EMB, and SQ109, as well as the fluroquinolone LEV. These results are consistent with previous findings that the inhibition of respiration by cotreating mycobacteria with respiratory inhibitors such as Q203 or BDQ attenuates the activity of INH, EMB, ETH, and moxifloxacin (another fluoroquinolone) by preventing a drug-induced lethal ATP burst (58–60). Similarly, disruptions to the NADH/NAD^+^ ratio by inhibition of NADH dehydrogenase activity also protect against INH killing (68, 84–87). Interestingly, other studies have demonstrated that inhibiting respiration through alternative mechanisms can potentiate INH killing in M. tuberculosis (14, 64), while both enhancing and inhibiting respiration can prevent the emergence of INH resistance (14, 63, 64). Despite this complexity, our results demonstrate a requirement for succinate oxidation in the susceptibility of M. tuberculosis to INH and other cell wall biosynthesis inhibitors under *in vitro* conditions.

Paradoxically, and in contrast to the above, when gene knockdown was induced simultaneously with antibiotic challenge, the knockdown of *sdhA1 *and* sdhA2* prevented the emergence of INH and PA-824 resistance. Interestingly, for PA-824 this phenotype was maintained in predepleted cells at high concentrations of PA-824, despite the attenuation of killing seen at lower concentrations. While our results indicate that predepletion of CRISPRi target enzymes is required to observe known chemical-genetic interactions, we suggest that the observation that impaired succinate oxidation prevents the emergence of INH and PA-824 resistance is relevant for several reasons, all of which may support our observations: (i) INH and PA-824 are both prodrugs whose mechanism of activation is linked to mycobacterial metabolism and respiration (86, 88, 89), (ii) both INH and PA-824 have relatively high frequencies of resistance compared to other TB drugs (89), and (iii) the development of both INH and PA-824 resistance can be prevented by inhibiting respiration in M. tuberculosis (14, 64). It is possible that the timing of the disruption to the ETC (i.e., prior to or simultaneously with INH or PA-824 treatment) affects the interactions observed (90), although this finding requires additional investigation.

Furthermore, our results highlight novel chemical-genetic interactions between ETC components that could be exploited for drug development. M. tuberculosis with both Sdh1 and Sdh2 transcriptionally depleted was hypersusceptible to growth inhibition and/or killing by other bioenergetic inhibitors, such as BDQ, TB47, and Q203. These findings add to previous reports of synergistic interactions between ETC complexes (13, 61, 62, 66, 69, 91–93) and support the proposal that targeting multiple components of mycobacterial energy generation could result in efficacious drug regimens (94).

Lastly, it is worth noting that while our results may appear to mimic the antibiotic susceptibility profile of M. tuberculosis under nonreplicating conditions (i.e., tolerant to conventional antibiotics but sensitive to respiration inhibitors), there are several key differences that demonstrate that this is not the case. Importantly, we did not observe attenuated killing for all bactericidal antibiotics tested (e.g., CFZ, THZ, STREP, and LZD), arguing against a general inhibition of killing by inducing bacteriostasis as has previously been reported (56). Further, all four of these antibiotics lose activity against nutrient-starved or hypoxic cultures (10, 95), whereas our data show equal susceptibility to killing by CFZ, THZ, STREP, and LZD in the presence or absence of Sdh1 and Sdh2. Additionally, our results showed a synergistic interaction with RIF at sub-MIC levels whereas RIF has reduced activity against nutrient-starved nonreplicating cultures (10, 39). These differences distinguish our findings from being a generic response to cells being in a nonreplicating state and instead argue that the interactions we observed are a specific response to impaired succinate oxidation in M. tuberculosis.

In summary, our work demonstrates that succinate oxidation is required for the optimal growth of M. tuberculosis and further defines the roles and essentiality of SDH/FRD enzymes in mycobacteria. The simultaneous transcriptional repression of *sdhA1 *and* sdhA2* significantly reduced the activity of the respiratory chain, had bacteriostatic consequences for cell viability, and influenced the susceptibility of M. tuberculosis to a variety of antibiotics. Combined, these findings provide useful insights into the physiology of energy metabolism of M. tuberculosis and its utility as a nascent area for antimicrobial development.

## MATERIALS AND METHODS

### Bacterial strains and culture conditions.

Escherichia coli strain MC1061 was used for the construction of all CRISPRi expression plasmids. E. coli MC1061 was grown at 37°C in liquid Luria-Bertani broth (LB) with agitation (200 rpm) or on solid Luria-Bertani agar (LBA) supplemented with 1.5% (wt/vol) agar. CRISPRi plasmids were selected for and maintained with 50 μg/mL kanamycin.

The M. tuberculosis H37Rv auxotrophic-attenuated derivative mc^2^6230 (Δ*RD1* Δ*panCD*) (96) was grown in Middlebrook 7H9 broth containing OADC (0.005% oleic acid, 0.5% bovine serum albumin [BSA] [Sigma], 0.2% dextrose, 0.085% catalase) and 0.05% tyloxapol. Medium was supplemented with 25 μg/mL pantothenic acid (PAN). For culturing M. tuberculosis mc^2^6230 on single carbon sources, a base of 7H9 liquid medium with 0.5% BSA, 0.085% NaCl, 0.05% tyloxapol, and 25 μg/mL PAN was supplemented with either glycerol (0.2%), glucose (20 mM), succinate (30 mM), acetate (0.2%), or a combination of acetate (0.1%) and glucose (10 mM). Cultures were maintained in 10-mL volumes in 30-mL inkwells at 37°C with agitation (140 rpm). CRISPRi plasmids were maintained in M. tuberculosis with 25 μg/mL kanamycin (KAN). When required, gene knockdown was induced by adding anhydrotetracycline (ATc) at 100 ng/mL. Solid medium was Middlebrook 7H11 agar plus OADC-PAN-KAN. For CFU determination, cultures were 10-fold serially diluted along a four-point dilution curve in 7H9, and 5 μL of each dilution was spotted onto 7H11 agar. Colonies were counted after 5 weeks of incubation at 37°C.

### Antimicrobial compounds.

Rifampicin (RIF), isoniazid (INH), streptomycin (STREP), clofazimine (CFZ), thioridazine (THZ), levofloxacin (LEV), pretomanid (PA-824), ethionamide (ETH), kanamycin (KAN), ATc, and SQ109 were purchased from Sigma-Aldrich. Linezolid (LZD) and ethambutol (EMB) were obtained from Selleck Chemicals. Bedaquiline (BDQ) was obtained from Toronto Research Chemicals. TB47 was a gift from Tianyu Zhang (Guangzhou Institutes of Biomedicine and Health, Chinese Academy of Sciences, China). Q203 and ND-011992 were gifts from Kevin Pethe (Lee Kong Chian School of Medicine, Nanyang Technological University, Singapore). All antibiotic stocks were prepared in dimethyl sulfoxide (DMSO), except for STREP, THZ, and KAN, which were prepared in H_2_O. ATc was dissolved in 70% ethanol.

### Construction of CRISPRi knockdown plasmids and strains.

For transcriptional knockdowns in M. tuberculosis, a 20- to 25-bp sequence downstream of a permissible protospacer-adjacent motif (PAM) sequence was identified in the nontemplate strand of *sdhA1* (Rv0248c), *sdhA2* (Rv3318), and *frdA* (Rv1553). The 20- to 25-bp sequences from the template and nontemplate strand were ordered as oligonucleotides with 5′ GGGA and AAAC overhangs, respectively. Information for individual sgRNAs (i.e., target sequence, PAM sequence, and predicted PAM strength) is provided in Table S1 in the supplemental material. Oligonucleotides used for plasmid construction are listed in Table S2. Oligonucleotides were annealed and cloned into CRISPRi plasmids (i.e., pJLR965) using BsmB1 Golden Gate cloning, sequence verified, and transformed into M. tuberculosis strain mc^2^6230 as previously described (48). Plasmids are listed in Table S3.

10.1128/mbio.01672-22.5 TABLE S1 sgRNA targeting *sdhA1*, *sdhA2*, *frdA*, and *cydB* in M. tuberculosis. Download Table S1, PDF file, 0.03 MB. Copyright © 2022 Adolph et al. 2022 Adolph et al. https://creativecommons.org/licenses/by/4.0/ This content is distributed under the terms of the Creative Commons Attribution 4.0 International license.

10.1128/mbio.01672-22.6 TABLE S2 Oligonucleotides used in this study. Download Table S2, PDF file, 0.03 MB. Copyright © 2022 Adolph et al. 2022 Adolph et al. https://creativecommons.org/licenses/by/4.0/ This content is distributed under the terms of the Creative Commons Attribution 4.0 International license.

10.1128/mbio.01672-22.7 TABLE S3 Plasmids used in this study. Download Table S3, PDF file, 0.03 MB. Copyright © 2022 Adolph et al. 2022 Adolph et al. https://creativecommons.org/licenses/by/4.0/ This content is distributed under the terms of the Creative Commons Attribution 4.0 International license.

Multiplexed plasmids that express two or three sgRNAs targeting a combination of *sdhA1*, *sdhA2*, and *frdA* were constructed by cloning additional sgRNAs into a SapI-based Golden Gate cloning site (46). Plasmids that express either a total of two or three sgRNAs were constructed using specific sets of primers to ensure that overhangs generated following SapI digestion would facilitate plasmid reassembly in a specific sgRNA order. In brief, multiplex plasmids that express a total of two sgRNAs were constructed by amplifying the additional sgRNA module (transcriptional promoter, sgRNA scaffold, and transcriptional terminator) from the appropriate CRISPRi plasmid using Phusion polymerase and the indicated primer pair (sgRNA-2 = MMO120 + MMO121) as described in Table S4. The amplified sgRNA module was purified and cloned into the CRISPRi plasmid that expresses the appropriate sgRNA partner using Golden Gate cloning with SapI, as described in Table S5. Multiplexed plasmids that express a total of three sgRNAs were constructed by amplifying the additional sgRNA modules from the appropriate CRISPRi plasmids as described above using the indicated primer pairs (sgRNA-2 = MMO120 + MMO123 and sgRNA-3 = MMO122 + MMO121). The amplified sgRNA modules were purified and cloned into the CRISPRi plasmid that expresses the appropriate sgRNA partner as described above. Constructed plasmids were sequence verified and transformed into M. tuberculosis strain mc^2^6230 as previously described (48). All constructed plasmids are listed in Table S3.

10.1128/mbio.01672-22.8 TABLE S4 Amplification of target sgRNA for multiplexed cloning. Download Table S4, PDF file, 0.03 MB. Copyright © 2022 Adolph et al. 2022 Adolph et al. https://creativecommons.org/licenses/by/4.0/ This content is distributed under the terms of the Creative Commons Attribution 4.0 International license.

10.1128/mbio.01672-22.9 TABLE S5 Multiplex golden gate cloning. Download Table S5, PDF file, 0.03 MB. Copyright © 2022 Adolph et al. 2022 Adolph et al. https://creativecommons.org/licenses/by/4.0/ This content is distributed under the terms of the Creative Commons Attribution 4.0 International license.

### RNA extraction and mRNA quantification by quantitative PCR (qPCR).

M. tuberculosis single and multiplexed *sdhA1*, *sdhA2*, and *frdA* CRISPRi strains were inoculated at an optical density at 600 nm (OD_600_) of 0.1 in 10 mL 7H9-OADC medium containing 100 ng/mL ATc. Cultures were harvested for RNA extraction after 3 days of gene knockdown as previously described (48). RNA was extracted using TRIzol reagent (Invitrogen) and purified using the RNA Clean and Concentrator kit (Zymo) as previously described (48). Samples were DNase treated using the Turbo DNA-free kit (Invitrogen) and confirmed to be DNA free by PCR using 1 μL of extracted RNA as a template with the primer combination of MMO200 and MMO201.

cDNA was synthesized from the RNA using the SuperScript IV Vilo master mix (Invitrogen) according to the manufacturer’s instructions. qPCRs were performed in 384-well plates in a ViiA7 Thermocycler using the Invitrogen PowerUp SYBR green master mix as previously described (48). Primer sequences are listed in Table S2. All qPCR primer pairs and cDNA masses tested were experimentally validated to be within the linear range of the assay. Signals were normalized to the housekeeping gene *sigA* and quantified by the threshold cycle (2^ΔΔ^*^CT^*) method.

### Nutrient starvation experiments.

The survival of M. tuberculosis knockdown strains under conditions of nutrient starvation was determined using previously published protocols (39). Briefly, M. tuberculosis knockdown strains were inoculated at a starting OD_600_ of 0.005 in complete medium (7H9 plus OADC-PAN-KAN) in the presence of 100 ng/mL ATc to induce CRISPRi and deplete cells of SDH and FRD enzymes. After 8 days, cultures were harvested and washed twice in PBS plus 0.05% tyloxapol. Cells were resuspended in PBS-tyloxapol and nutrient starved by inoculation into inkwells containing PBS-tyloxapol, KAN, and 100 ng/mL ATc at an OD_600_ of 0.01 (i.e., approximately 10^5^ CFU/mL). PAN was not included as M. tuberculosis mc^2^6230 has previously been shown not to require PAN under conditions of nutrient starvation (97, 98). Cultures were incubated at 37°C without shaking for 8 weeks, and viability was measured by plating for CFU every 7 days.

### Oxygen consumption rate measurements.

Oxygen consumption rate measurements of exponentially growing M. tuberculosis cells were performed as previously described using an Oroboros O2k FluoRespirometer (61, 67). In brief, M. tuberculosis strains expressing sgRNA targeting *sdhA1*, *sdhA2*, and *frdA* in single or multiplexed constructs were inoculated at a starting OD_600_ of 0.005 in 10 mL 7H9 medium supplemented with either OADC, glycerol, or succinate and 0 or 100 ng/mL ATc. Cultures were grown for 8 days until the *sdhA1 *+* sdhA2* double knockdown strain reached an OD_600_ of ~0.2. All cultures were adjusted to an OD_600_ of 0.2 in supplemented 7H9 medium on the day of experimentation from log-phase cultures. Two milliliters of culture was added to each measurement chamber, which contains an O_2_-sensing Clark-type electrode. Oxygen consumption rates were monitored for 15 min once oxygen consumption reached a steady state. All measurements were made at 37°C with 750 rpm in a closed chamber and a data recording interval of 2 s^−1^. The combined inhibition of both terminal oxidases with Q203 (400 nM) and ND-011992 (100 μM) was used to demonstrate complete inhibition of oxygen consumption. Chemicals were added after 10 min of respiration at a steady state through the injection port of the stoppers using Hamilton syringes. Oxygen consumption rates were calculated using the Oroboros Data Lab software.

### Antibiotic susceptibility testing.

The MICs and minimum bactericidal concentrations (MBCs) of the M. tuberculosis *sdhA1 *+* sdhA2* CRISPRi strain were determined as previously described (48). In brief, M. tuberculosis was inoculated into a 96-well plate containing 7H9 plus OADC-PAN-KAN medium at an OD_600_ of 0.005 in a final volume of 150 μL with 0 or 100 ng/mL ATc. Antibiotics were dispensed from a 9-point, 3-fold dilution gradient into each well, with a maximum of 2% DMSO. Cultures were incubated at 37°C for 10 days without agitation. On day 10, OD_600_ values were measured in a Varioskan Lux microplate reader and MIC values for the no-knockdown controls were calculated using the Gompertz equation (99). For MBC determination, cultures were removed from rows containing 0 and 100 ng/mL ATc at day 10 and 10-fold serially diluted in 7H9 and 5 μL of each dilution was spotted onto 7H11 agar. Colonies were counted after 5 weeks of incubation at 37°C.

For preknockdown experiments, the M. tuberculosis *sdhA1 *+* sdhA2* CRISPRi strain was grown in 7H9-OADC-PAN-KAN medium in 10-mL volumes from a starting OD_600_ of 0.005 for 6 days prior to antibiotic challenge. Cultures were treated with 0 or 100 ng/mL ATc on day 0 to induce gene silencing. After 6 days, cultures were inoculated at an OD_600_ of 0.005 into a 96-well plate containing 7H9 plus OADC-PAN-KAN medium, 0 or 100 ng/mL ATc, and a 9-point, 3-fold dilution gradient of the respective antibiotic. Plates were incubated for 10 days before plating for CFU per milliliter as described above.

### Time-kill experiments.

M. tuberculosis cultures were inoculated into inkwells containing 10 mL 7H9 medium supplemented with OADC-PAN-KAN at a starting OD_600_ of 0.005. Cultures were treated with 0 or 100 ng/mL ATc and with selected antibiotics at the specified concentrations. Bacterial viability was determined by plating onto 7H11 agar plates at specified time points and enumerating CFU after 5 weeks of incubation at 37°C. For preknockdown experiments, strains were incubated in the presence of 0 or 100 ng/mL ATc for 6 days as described above before being inoculated into inkwells.
