# Supplementary figures and images for: Impaired Succinate Oxidation Prevents Growth and Influences Drug Susceptibility in Mycobacterium tuberculosis
Source: mBio. 2022 Jul 20;13(4):e01672-22. doi: 10.1128/mbio.01672-22 (PMC9426501; doi:10.1128/mbio.01672-22)

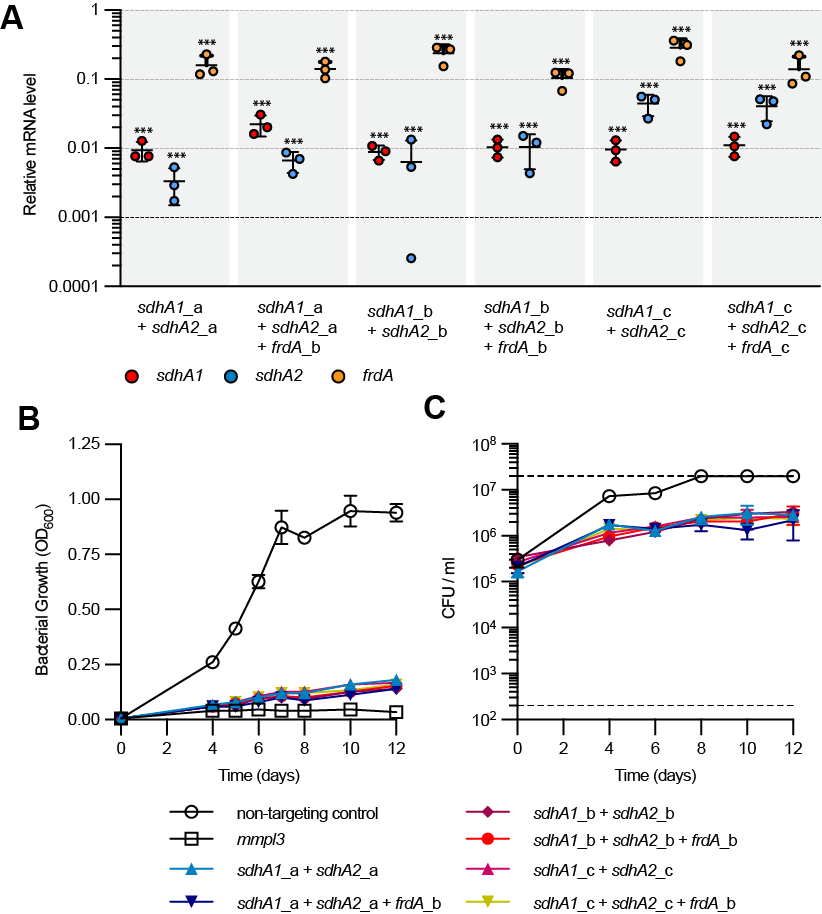

Supplement: FIG S1 [file mbio.01672-22-s0001.tif]

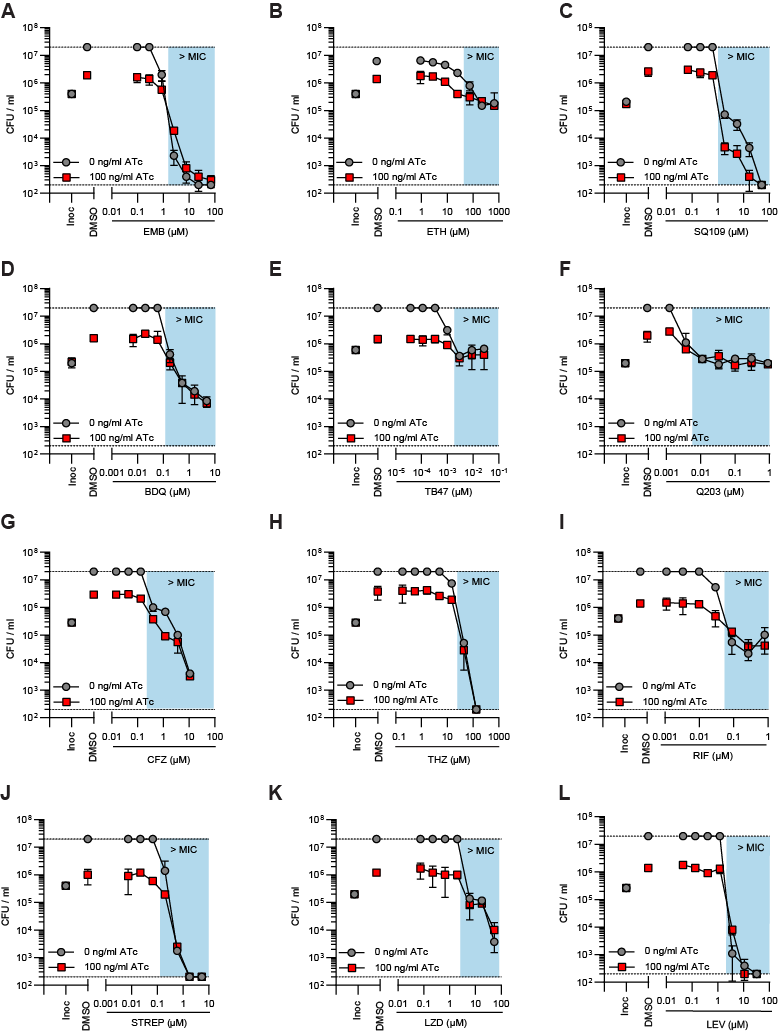

Supplement: FIG S2 [file mbio.01672-22-s0002.tif]

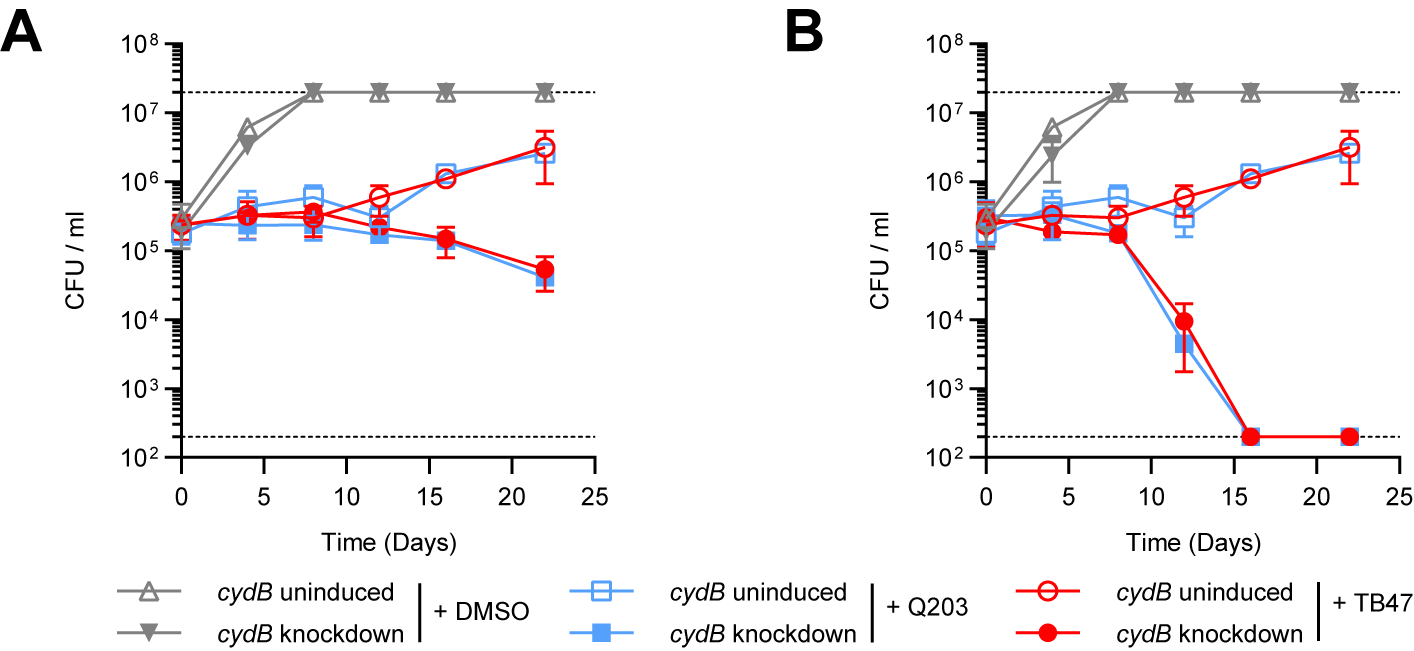

Supplement: FIG S3 [file mbio.01672-22-s0003.tif]

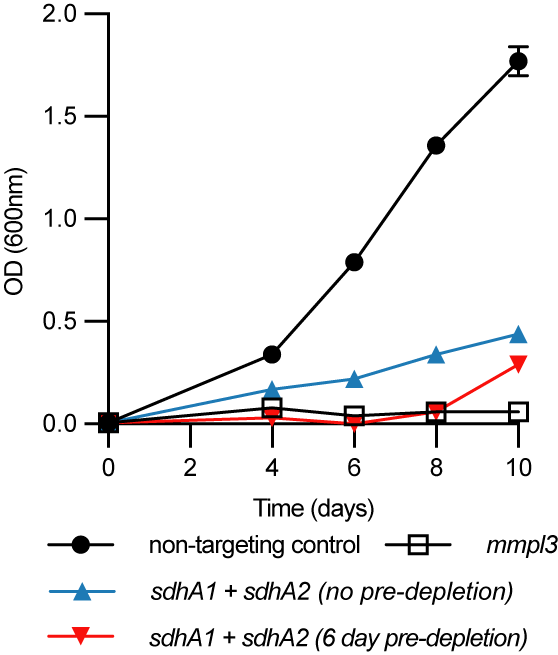

Supplement: FIG S4 [file mbio.01672-22-s0004.tif]
