# Supplementary material for: Impaired Succinate Oxidation Prevents Growth and Influences Drug Susceptibility in Mycobacterium tuberculosis
Source: mBio. 2022 Jul 20;13(4):e01672-22. doi: 10.1128/mbio.01672-22 (PMC9426501; doi:10.1128/mbio.01672-22)
Supplement: TABLE S1 [file mbio.01672-22-s0005.pdf]

Table S1: sgRNA targeting *sdhA1*, *sdhA2*, *frdA* and *cydB* in *M. tuberculosis*

Target	sgRNA name	PAM sequence (5'-3' direction on the template strand)	PAM score [a]	Targeted sequence in target gene (5'-3' direction on the non-template strand)
Rv1552	frdA_a	ACAGAAT	2	GCACCGGCGGATGCGGGCGGGT
Rv1552	frdA_b	TTGGAAA	6	GGTGTACCCGATGCGCAGCC
Rv1552	frdA_c	GCAGGAG	8	ACACGTTGTTTCAACGGCTGC
Rv0248c	sdhA1_a	CCAGAAT	2	TCACTGAGGGTGTTCGCGGC
Rv0248c	sdhA1_b	GTAGGAG	5	GACGTAGTCGTGATCGGTGC
Rv0248c	sdhA1_c	TCAGGAA	14	ACAACTGGCGTATGGCCGAGC
Rv3318	sdhA2_a	GCAGAAT	2	AGACGCTGTATCAGAACTGC
Rv3318	sdhA2_b	GAAGAAC	5	AACGAGTTTTACGCGCTGGAT
Rv3318	sdhA2_c	AGGGAAG	4	TGGAGGACATGGAGTTTCAC
Rv1622c	cydB_a	GAAGAAA	3	ATCCTAGAAGGGTTCGACTT

[a]: PAM score as ranked out of 15 possible permissible sequences (46)
