# Supplementary material for: Impaired Succinate Oxidation Prevents Growth and Influences Drug Susceptibility in Mycobacterium tuberculosis
Source: mBio. 2022 Jul 20;13(4):e01672-22. doi: 10.1128/mbio.01672-22 (PMC9426501; doi:10.1128/mbio.01672-22)
Supplement: TABLE S2 [file mbio.01672-22-s0006.pdf]

Table S2: Oligos used in this study

Name	Sequence	Description
**CRISPRi cloning oligos**		
FrdA_TB_Fcs	GGGAACCCGCCCGCATCCGCCGGTGC	Fwd cloning oligo for CRISPRi
FrdA_TB_Rcs	AAACGCACCGGCGGATGCGGGCGGGT	Rev cloning oligo for CRISPRi
FrdAb_TB_Fcs	GGGAGGCTGCGCATCGGGTACACC	Fwd cloning oligo for CRISPRi
FrdAb_TB_Rcs	AAACGGTGTACCCGATGCGCAGCC	Rev cloning oligo for CRISPRi
FrdAc_TB_Fcs	GGGAGCAGCCGTTGAAACAACGTGT	Fwd cloning oligo for CRISPRi
FrdAc_TB_Rcs	AAACACACGTTGTTTCAACGGCTGC	Rev cloning oligo for CRISPRi
SdhA1_TB_Fcs	GGGAGCCGCGAACACCCTCAGTGA	Fwd cloning oligo for CRISPRi
SdhA1_TB_Rcs	AAACTCACTGAGGGTGTTCGCGGC	Rev cloning oligo for CRISPRi
SdhA1b_TB_Fcs	GGGAGCACCGATCACGACTACGTC	Fwd cloning oligo for CRISPRi
SdhA1b_TB_Rcs	AAACGACGTAGTCGTGATCGGTGC	Rev cloning oligo for CRISPRi
SdhA1c_TB_Fcs	GGGAGCTCGGCCATACGCCAGTTGT	Fwd cloning oligo for CRISPRi
SdhA1c_TB_Rcs	AAACACAACTGGCGTATGGCCGAGC	Rev cloning oligo for CRISPRi
SdhA2_TB_Fcs	GGGAGCAGTTCTGATACAGCGTCT	Fwd cloning oligo for CRISPRi
SdhA2_TB_Rcs	AAACAGACGCTGTATCAGAACTGC	Rev cloning oligo for CRISPRi
SdhA2b_TB_Fcs	GGGAATCCAGCGCGTAAAACTCGTT	Fwd cloning oligo for CRISPRi
SdhA2b_TB_Rcs	AAACAACGAGTTTTACGCGCTGGAT	Rev cloning oligo for CRISPRi
SdhA2c_TB_Fcs	GGGAGTGAAACTCCATGTCCTCCA	Fwd cloning oligo for CRISPRi
SdhA2c_TB_Rcs	AAACTGGAGGACATGGAGTTTCAC	Rev cloning oligo for CRISPRi
cydB_a_TB_Fcs	GGGAAAGTCGAACCCTTCTAGGAT	Fwd cloning oligo for CRISPRi
cydB_a_TB_Fcs	AAACATCCTAGAAGGGTTCGACTT	Rev cloning oligo for CRISPRi
**qPCR oligos**		
MMO33	AGCCATCGAGCTGGGATTTT	SdhA2_TB Fwd qPCR primer
MMO32	CCTTGTAGGCCATGGTGTGT	SdhA2_TB Rev qPCR primer
MMO149	TACGCGCTGCATATGGACTT	SdhA1_TB Fwd qPCR primer
MMO150	ACGAGTACGTTGTGGTAGCG	SdhA1_TB Rev qPCR primer
MMO153	GACGACGAGCATTTCTTGGC	FrdA_TB Fwd qPCR primer
MMO154	CCAGCGAGTGATAGTGACCG	FrdA _TB Rev qPCR primer
MMO173	GAGGAGATCGCTGAACCCAC	SigA_TB Fwd qPCR primer
MMO174	CTGTTTGAGGTAGGCGCGAA	SigA_TB Rev qPCR primer
**Multiplex cloning oligos (Red denotes the sap1 cut site, blue is the generated overhang)**		
MMO120	AATATGCTCTTCAGGATCTGACCAGGGAA AATAGCC	Fwd primer for amplification of sgRNA modules
MMO121	TTTATGCTCTTCACTGAAAAAAAAAACACC CTGCCATAAAATGAC	Rev primer for amplification of sgRNA modules
MMO122	AATATGCTCTTCAGCTTCTGACCAGGGAA AATAGCC	Fwd primer for amplification of sgRNA modules

MMO123	TTTATGCTCTTCAAGCAAAAAAAAAACACC CTGCCATAAAATGAC	Rev primer for amplification of sgRNA modules
