# Supplementary material for: Impaired Succinate Oxidation Prevents Growth and Influences Drug Susceptibility in Mycobacterium tuberculosis
Source: mBio. 2022 Jul 20;13(4):e01672-22. doi: 10.1128/mbio.01672-22 (PMC9426501; doi:10.1128/mbio.01672-22)
Supplement: TABLE S3 [file mbio.01672-22-s0007.pdf]

Table S3: Plasmids used in this study

Name	Description	Plasmid Backbone	Reference
pJLR965	CRISPRi cloning vector for *M. tuberculosis*- vector expressing dCas9 and non-targeting sgRNA		(46)
**Single sgRNA plasmids**			
pCi7	CRISPRi vector expressing dCas9 + sgRNA targeting *mmpL3*	pJLR965	(48)
pCi5	CRISPRi vector expressing dCas9 + sgRNA (*frdA*_a) targeting *frdA*	pJLR965	This study
pCi8	CRISPRi vector expressing dCas9 + sgRNA (*sdhA1*_a) targeting *sdhA1*	pJLR965	(42)
pCi9	CRISPRi vector expressing dCas9 + sgRNA *(sdhA2*_a) targeting *sdhA2*	pJLR965	(42)
pCi23	CRISPRi vector expressing dCas9 + sgRNA *(frdA*_b) targeting *frdA*	pJLR965	(42)
pCi24	CRISPRi vector expressing dCas9 + sgRNA (*frdA*_c) targeting *frdA*	pJLR965	(42)
pCi25	CRISPRi vector expressing dCas9 + sgRNA (*sdhA1*_b) targeting *sdhA1*	pJLR965	(42)
pCi26	CRISPRi vector expressing dCas9 + sgRNA (*sdhA1*_c) targeting *sdhA1*	pJLR965	This study
pCi27	CRISPRi vector expressing dCas9 + sgRNA (*sdhA2*_b) targeting *sdhA2*	pJLR965	(42)
pCi28	CRISPRi vector expressing dCas9 + sgRNA (*sdhA2*_c) targeting *sdhA2*	pJLR965	This study
pCi95	CRISPRi vector expressing dCas9 + sgRNA (*cydB*) targeting *cydB*	pJLR965	(42)
**Multiplexed plasmids**			
pCiMX15	CRISPRi vector expressing dCas9 + sgRNAs (*sdhA1*_a and *sdhA2*_a)	pCi8	This study
pCiMX22	CRISPRi vector expressing dCas9 + sgRNAs (*frdA*_b and s*dhA1*_a)	pCi23	This study
pCiMX23	CRISPRi vector expressing dCas9 + sgRNAs (*frdA*_b and s*dhA2*_a)	pCi23	This study
pCiMX24	CRISPRi vector expressing dCas9 + sgRNAs (*frdA*_b, *sdhA1*_a and *sdhA2_)*	pCi23	This study
pCiMX30	CRISPRi vector expressing dCas9 + sgRNAs (*sdhA1*_b and *sdhA2*_b)	pCi25	This study
pCiMX31	CRISPRi vector expressing dCas9 + sgRNAs (*frdA*_b, *sdhA1*_b and *sdhA2*_b)	pCi23	This study
pCiMX34	CRISPRi vector expressing dCas9 + sgRNAs (*sdhA1*_c and *sdhA2*_c)	pCi26	This study
pCiMX35	CRISPRi vector expressing dCas9 + sgRNAs (*frdA*_c, *sdhA1*_c and *sdhA2*_c)	pCi24	This study
