# Supplementary material for: Impaired Succinate Oxidation Prevents Growth and Influences Drug Susceptibility in Mycobacterium tuberculosis
Source: mBio. 2022 Jul 20;13(4):e01672-22. doi: 10.1128/mbio.01672-22 (PMC9426501; doi:10.1128/mbio.01672-22)
Supplement: TABLE S4 [file mbio.01672-22-s0008.pdf]

Table S4: Amplification of target sgRNA for multiplexed cloning

**Component**	**50 µl RxN (µl)**		
H_2O	31		
5x Phusion GC Buffer	10		
10 mM dNTPs	1		
Fwd Primer (10 µM stock)	2.5		
Rev Primer (10 µM stock)	2.5		
pCi plasmid with target sgRNA	1 µl		
DMSO	1.5		
Phusion Poln	0.5		
Total	50		
**Thermocycler instructions**			
**Step**	**Temp**	**Time**	**Cycles**
Initial Denaturation	98 °C	30 sec	1
Denaturation	98 °C	10 sec	35
Annealing	60 °C	30 sec	
Extension	72 °C	1 min	
Final Extension	72 °C	10 min	1
Hold	4 °C	hold	
