# Supplementary material for: Impaired Succinate Oxidation Prevents Growth and Influences Drug Susceptibility in Mycobacterium tuberculosis
Source: mBio. 2022 Jul 20;13(4):e01672-22. doi: 10.1128/mbio.01672-22 (PMC9426501; doi:10.1128/mbio.01672-22)
Supplement: TABLE S5 [file mbio.01672-22-s0009.pdf]

Table S5: Multiplex golden gate cloning

Component	Volume (µl)	
10× T4 Ligase Buffer	1	
pCi uncut (20 ng/µL)	1.25	
Cloned and purified sgRNA	2.5	
Sap1 (10,000u/ml)	0.5	
T4 DNA Ligase	0.5	
mQ	Up to 10 µl total volume	
**Temperature (℃)**	**Duration**	
37 (digestion)	5 min	30 cycles
16 (ligation)	5 min	
4	Forever	1
